# Chronic disease and sitting time in middle-aged Australian males: findings from the 45 and Up Study

**DOI:** 10.1186/1479-5868-10-20

**Published:** 2013-02-08

**Authors:** Emma S George, Richard R Rosenkranz, Gregory S Kolt

**Affiliations:** 1 School of Science and Health, University of Western Sydney, Sydney, Australia; 2 Department of Human Nutrition, Kansas State University, Manhattan, KS, U.S.A

**Keywords:** Physical activity, Sedentary behaviour, Sedentary lifestyle, Chronic disease, Heart disease, Cancer, Diabetes, Blood pressure

## Abstract

**Background:**

Compared to females, males experience a range of health inequities including higher rates of diabetes and cardiovascular disease. Although sitting time is emerging as a distinct risk factor for chronic disease, research on the association of sitting time and chronic disease in middle-aged Australian males is limited.

**Methods:**

A sample of 63,048 males aged 45-64 years was drawn from the baseline dataset of the 45 and Up Study – a longitudinal cohort study on healthy ageing with 267,153 participants from across New South Wales, Australia’s most populous state. Baseline data on self-reported chronic disease (heart disease, cancer, diabetes, high blood pressure, combined chronic diseases), sitting time, physical activity (Active Australia Survey), and a range of covariates were used for cross-sectional analyses. Crude (OR), partially and fully adjusted odds ratios (AOR) and 95% confidence intervals (CI) were calculated using binary logistic regression.

**Results:**

Compared to those sitting <4 hours/day, participants reporting 4 to <6, 6 to <8, and ≥8 hours were significantly more likely to report ever having any chronic disease (AOR 1.06, 95% CI 1.00 – 1.12, p = 0.050; AOR 1.10, 95% CI 1.03 – 1.16, p = 0.003; AOR 1.09, 95% CI 1.03 – 1.15, p = 0.002, respectively). Participants who reported 6 to <8 hours and ≥8 hours of sitting were also significantly more likely to report ever having diabetes than those reporting <4 hours/day (AOR 1.15, 95% CI 1.03 – 1.28, p = 0.016; AOR 1.21, 95% CI 1.09 – 1.33, p <0.001, respectively).

**Conclusions:**

Our findings suggest that higher volumes of sitting time are significantly associated with diabetes and overall chronic disease, independent of physical activity and other potentially confounding factors. Prospective studies using valid and reliable measures into domain-specific sitting time in middle-aged males are required to understand and explain the direction of these relationships.

## Background

Research into the area of male health is gaining momentum in countries across the world, and has been highlighted by the release of a range of male-specific health reports and policies [1-4]. Australian males experience higher rates of a range of chronic diseases, such as diabetes and cardiovascular disease (CVD), in comparison to their female counterparts [1]. Australian data from 2007 showed that cancer and other tumours were the leading cause of death in both males and females aged between 45 and 64 years, while CVD, including both coronary heart disease (CHD) and stroke, was the second highest cause of death in this age group [5].

It has been well established that participation in regular physical activity has the potential to reduce a person’s risk of developing various chronic diseases [6,7]. Among middle-aged and older males, specifically, physical activity has been found to be inversely associated with CHD risk [8,9], hypertension [8], cancer mortality [10], and CVD mortality [11]. Further, greater leisure time physical activity (LTPA), has been associated with reduced diabetes risk [12], while high lifetime occupational physical activity has been shown to be protective against colon and prostate cancer in adult males [13].

Researchers have established that physical (in)activity and sedentary behaviour are two distinct risk factors that can independently affect health. Sedentary behaviour is characterised by activities such as sitting or lying down, involving energy expenditure of 1.0-1.5 metabolic equivalents [14]. Independent of LTPA, higher levels of daily sitting time have been found to increase the risk of both CVD [15] and all-cause mortality in adults [15,16]. Specific (presumably) sedentary behaviours such as television viewing have also been associated with higher CVD mortality risk in males [11], increased likelihood of having the metabolic syndrome [17] and increased diabetes risk [12]. Time spent in sedentary behaviours has also been associated with clustered metabolic risk, independent of physical activity [18]. In addition to these findings that have demonstrated associations between sedentary time and health outcomes, other prospective studies have provided evidence of reverse causality, whereby specific health indicators such as body mass index (BMI) have been shown to predict sedentary time [19,20].

Current literature highlights the importance of participating in regular physical activity and limiting sedentary time for positive health outcomes. A number of studies have focused on specific domains of sedentary behaviour (e.g. television viewing), however, it has been demonstrated that television viewing time is not necessarily representative of overall sedentary time [21,22]. Furthermore, although several studies have examined the association between sedentary time and specific chronic diseases, few have examined overall time spent sitting on the association with a range of chronic diseases, particularly in middle-aged males.

The aim of this study was to build upon an existing and growing body of literature on sitting time – and the association of this modifiable lifestyle behaviour – with a range of chronic diseases. This study utilised a large sample of middle-aged Australian males – a relatively understudied population group – and statistically controlled for a range of associated covariates, including age, BMI, and functional limitation.

## Methods

### The 45 and Up Study

The 45 and Up Study has been described in detail elsewhere [23]. Briefly, the 45 and Up Study is a large-scale Australian cohort study of 267,153 individuals from across New South Wales (NSW), the most populous state in Australia. Data derived from the 45 and Up Study baseline questionnaire [24] provide insight into an extensive range of health conditions and underlying determinants of health. Participants were randomly sampled from the Medicare Australia (national health insurance) database between February 2006 and December 2008. All adults who were aged 45 years and over and who were currently residing in NSW at the time of recruitment were eligible for inclusion in the Study. Participants were included in the Study if they completed a mailed baseline questionnaire and provided their signed consent for participation in the baseline questionnaire and long-term follow-up [23].

Ethics approval for the 45 and Up Study and analysis of the baseline questionnaire data was granted by the University of NSW Human Research Ethics Committee (approval number 05035). The University of Western Sydney Human Research Ethics Committee granted reciprocal ethics approval for use of the baseline questionnaire data in the current study (UWS Protocol number H8793).

### Participants

Participants were a subset of males aged between 45 and 64 years of age from the total 267,153 males and females enrolled in the 45 and Up Study as of December 2009 (18% response rate). A total of 123,799 study participants were male, and 70,416 of these were aged between 45 and 64 years (26.4% of the total sample). The final sample size for the current study was 63,048 males, after excluding those with missing or invalid data for two or more types of physical activity (walking, moderate physical activity, and vigorous physical activity; n = 7,368).

### Data

Data on all variables included in this analysis were derived from self-report measures from the 45 and Up Study baseline questionnaire.

Participants were asked to report whether they had ever been told by a doctor that they have a chronic disease or condition (question 24 of the 45 and Up Study baseline questionnaire). For the purpose of the current study, cancer (which included prostate cancer and other cancers, but did not include melanoma or non-melanoma skin cancer), heart disease, diabetes, and hypertension were used as primary outcome variables (binary presence or absence of each disease), as evidence suggests that time spent sedentary is associated with these, and other conditions [12,15,17,18]. In addition, an overall chronic disease variable combining the diseases listed above was computed (binary presence or absence of any chronic disease, among those listed) and analysed in a separate model.

Total sitting time was determined by asking participants to record the total amount of time, in hours, they usually spent sitting per day, and values were divided into quartiles of 0 to <4 hours, 4 to <6 hours, 6 to <8 hours, and ≥8 hours of sitting time per day. This particular question has not been assessed for reliability and validity, although it is analogous to the sitting time assessment question used in the International Physical Activity Questionnaire (IPAQ). Participants completing the IPAQ are asked to report on the amount of time, in hours and minutes, they usually spend sitting on a daily basis, and this item used to assess sedentary time has been shown to have acceptable reliability and validity [25]. Clemes, David, Zhao, Han, and Brown [26] found that compared with accelerometer data, a single-item question assessing overall sitting time significantly underestimated sitting time, while a multiple-question domain-specific questionnaire showed increased accuracy. Nevertheless, Atkin et al. [27], support the use of single-item questionnaires in health-related epidemiological research, suggesting that such tools are appropriate when the primary requirements include usability and the capability to rank behaviours of interest.

The Active Australia Survey (AAS) [28], which has been shown to have acceptable test-retest reliability [29] and validity [30], was used to measure physical activity in the 45 and Up Study baseline questionnaire. Participants were asked to report on their participation in three types of physical activity, with a reference period of the previous week (questions 16 and 17 of the 45 and Up Study baseline questionnaire) – “walking continuously, for at least 10 minutes (for recreation or exercise or to get to or from places)”; “vigorous physical activity (that made you breathe harder or puff and pant, like jogging, cycling, aerobics, competitive tennis, but not household chores or gardening)”; and “moderate physical activity (like gentle swimming, social tennis, vigorous gardening or work around the house)” – by recording the total duration and the total number of times they participated in each [28]. For the current study, the total time spent in these activities was used to determine participants’ physical activity levels, with minutes of vigorous physical activity given double weighting [28].

According to the World Health Organization (WHO) [6], 150 minutes or more of moderate-intensity and/or vigorous-intensity physical activity on a weekly basis is conducive to gaining health benefits in adults aged between 18 and 64 years. A total of five distinct physical activity categories were established for this study: 0 (no physical activity); 1 to 149 minutes of physical activity (insufficient levels); 150 to 299 minutes of physical activity (sufficient levels); 300 to 539 minutes (highly active); and ≥540 minutes (very highly active) of physical activity in the previous week.

Highest educational qualification was self-reported, with options including no qualifications, school or higher school certificate, trade or apprenticeship, certificate or diploma, and university degree. Participants also selected the most appropriate of nine categories for their pre-tax household income. For this study, these categories were combined to form five income categories (less than $10,000, $10,000-$29,999 per year, $30,000-$49,999 per year, $50,000-$69,999 per year, $70,000 or more per year). All other values were coded as missing. Questionnaire respondents indicated whether they had been a regular smoker in the past, and smoking status was categorised as ‘ever’ or ‘never’.

BMI (kg/m^2^) was calculated from participants’ self-reported height and weight measurements, and cut-points developed by the WHO were used to determine underweight (<18.50kg/m^2^), normal weight (18.50–24.99kg/m^2^), overweight (25.00–29.99kg/m^2^) and obese (≥30.00kg/m^2^) categories [31]. Functional limitation was measured using the Medical Outcomes Study Physical Functioning (MOS-PF) scale, which assesses the extent to which an individuals’ health limits their ability to perform daily functional activities [32]. Functional limitation scores (out of 100) were divided into 4 categories: no limitation (100), minor limitation (95–99), moderate limitation (85–94) and severe limitation (0–84). The MOS-PF has been shown to have good test-retest reliability and content validity as a measure of physical functioning [33].

### Statistical methods

Data drawn from the 45 and Up Study baseline dataset were analysed using SPSS 18.0 statistical software (SPSS Inc. Chicago, IL USA). To explore the demographic characteristics of the study sample, frequencies were calculated for all outcome and exposure variables, and covariates. Binary logistic regression analyses were used to examine the odds of having each (and also any) chronic disease by categories of sitting time, with partially adjusted models controlling for physical activity, age group, educational qualification, pre-tax household income, smoking status, and BMI categories, and fully adjusted models also controlling for functional limitation. Results are presented as crude odds ratios (OR), and partially and fully adjusted odds ratios (AOR) and corresponding 95% confidence intervals (CI). Unless otherwise specified, results refer to the fully adjusted odds ratios (AOR). A significance level of alpha = 0.05 was used in all analyses.

## Results

The mean ± standard deviation (SD) age of participants included in the current study was 55.6 (± 5.4 years), and the mean ± SD BMI was 27.6 (± 4.3 kg/m^2^). The demographic information for the sample is presented in Table 1. Of the 63,048 males included in this analysis, 41.3% reported ever having at least one chronic disease, 5.6% reported ever having cancer, 8.6% reported ever having heart disease, 7.7% reported ever having diabetes, and 31.3% reported ever having high blood pressure. A total of 80.9% of males reported participating in at least 150 minutes of physical activity in the previous week, while a total of 4.1% of males reported no physical activity. Eight or more hours of sitting per day was recorded by 32.8% of participants within the current sample. A total of 71.9% of the participants were overweight or obese, 41.9% had no functional limitation, 43.4% reported a pre-tax household income of ≥$70,000, and over 50% had either obtained a certificate or diploma (20.8%) or university degree (31.4%). A similar proportion of participants reported either ‘ever’ being a regular smoker (48.4%) or ‘never’ being a regular smoker (51.6%).

**Table 1:** Demographic characteristics of the sample

**Characteristics**	**Total**	
	**N**	**%**
**Chronic diseases**		
None	37,013	58.7
One	19,550	31.0
Two or more	6,485	10.3
Cancer	3,528	5.6
Heart disease	5,447	8.6
Diabetes	4,872	7.7
High blood pressure	19,753	31.3
**Sitting (hours/day)**		
0 to <4	14,079	23.3
4 to <6	15,369	25.4
6 to <8	11,255	18.6
≥8	19,847	32.8
**Physical activity**		
Sedentary (Zero)	2,571	4.1
Low active (1-149 mins)	9,505	15.1
Sufficiently active (150-299 mins)	10,712	17.0
Highly active (300-539 mins)	14,047	22.3
Very highly active (540+ mins)	26,213	41.6
**Age**		
45 to 49	12,427	19.7
50 to 54	16,142	25.6
55 to 59	17,940	28.5
60 to 64	16,539	26.2
**Pre-tax household income (AUD)**		
<10k	1,890	3.4
10k to <30k	7,494	13.5
30k to <50k	9,549	17.2
50k to <70k	9,183	16.5
70k +	27,378	49.3
**Educational qualification**		
None	4,368	7.0
School Certificate	7,972	12.8
HSC	6,502	10.4
Trade/Apprenticeship	10,748	17.2
Certificate/Diploma	13,109	21.0
University degree	19,782	31.7
**Smoking status**		
Never	32,520	51.6
Ever	30,496	48.4
**Body Mass Index**		
Underweight	329	0.5
Normal weight	16,477	27.5
Overweight	28,574	47.8
Obese	14,447	24.1
**Functional limitation**		
No limitation	26,442	44.5
Minor limitation	12,181	20.5
Moderate limitation	10,012	16.9
Severe limitation	10,731	18.1

### Combined chronic diseases

Males who reported sitting for 4 to <6 (OR 1.09, 95% CI 1.04 – 1.14, p <0.001) and 6 to <8 hours per day (OR 1.12, 95% CI 1.07 – 1.18, p <0.001) were significantly more likely to report ever having a chronic disease (Table 2), while those in the highest quartile (≥8 hours per day) were not significantly more likely to report ever having a chronic disease than those in the reference category of <4 hours of sitting time (OR 1.04, 95% CI 0.99 – 1.08). Across quartiles, there was a significant trend between sitting time and chronic disease (*P for trend* <0.001). Partial adjustment for covariates strengthened the association between sitting time and chronic disease, and males in all sitting categories higher than the reference category were significantly more likely to report ever having a chronic disease (*P for trend* <0.001). Once functional limitation was added to the fully adjusted model, the odds of having a chronic disease were attenuated, but remained significant (*P for trend* = 0.008), and males in each quartile above the reference category were significantly more likely to report ever having a chronic disease than those sitting for <4 hours per day (AOR 1.06, 95% CI 1.00 – 1.12, p = 0.050; AOR 1.10, 95% CI 1.03 – 1.16, p = 0.003; AOR 1.09, 95% CI 1.03 – 1.15, p = 0.002, respectively).

**Table 2:** Presence of chronic disease by categories of sitting time

**Odds (95% CI) of having a chronic disease**										
	**Any chronic disease**		**Cancer**		**Heart disease**		**Diabetes**		**High blood pressure**	
**Sitting time model 1**^**a**^										
0 to <4^*^	1.00		1.00		1.00		1.00		1.00	
4 to <6	1.09	1.04 - 1.14	1.07	0.97 - 1.18	1.07	0.99 - 1.16	1.12	1.03 - 1.23	1.06	1.01 - 1.11
6 to <8	1.12	1.07 - 1.18	1.14	1.02 - 1.27	1.13	1.03 - 1.23	1.19	1.08 - 1.31	1.10	1.04 - 1.16
≥8	1.04	0.99 - 1.08	0.98	0.89 - 1.08	0.95	0.88 - 1.03	1.15	1.06 - 1.25	1.04	0.99 - 1.09
**Sitting time model 2**^**b**^										
0 to <4^*^	1.00		1.00		1.00		1.00		1.00	
4 to <6	1.07	1.01 - 1.13	1.03	0.92 - 1.16	1.03	0.94 - 1.14	1.04	0.94 - 1.16	1.04	0.98 - 1.11
6 to <8	1.12	1.06 - 1.19	1.13	1.00 - 1.27	1.13	1.02 - 1.25	1.18	1.05 - 1.32	1.08	1.01 - 1.15
≥8	1.12	1.06 - 1.19	1.07	0.95 - 1.19	1.04	0.95 - 1.15	1.25	1.13 - 1.38	1.09	1.03 - 1.15
**Sitting time model 3**^**c**^										
0 to <4^*^	1.00		1.00		1.00		1.00		1.00	
4 to <6	1.06	1.00 - 1.12	1.02	0.91 - 1.15	1.01	0.92 - 1.11	1.03	0.92 - 1.14	1.03	0.97 - 1.09
6 to <8	1.10	1.03 - 1.16	1.10	0.97 - 1.24	1.08	0.98 - 1.20	1.15	1.03 - 1.28	1.06	0.99 - 1.13
≥8	1.09	1.03 - 1.15	1.03	0.92 - 1.16	0.99	0.90 - 1.08	1.21	1.09 - 1.33	1.06	1.00 - 1.12

* Reference category.

^a^ Unadjusted odds for sitting time alone.

^b^ Adjusted for physical activity, age, pre-tax household income, educational qualification, smoking status, and BMI.

^c^ Adjusted for physical activity, age, pre-tax household income, educational qualification, smoking status, BMI and functional limitation.

### Cancer

Sitting time was significantly associated with cancer (*P for trend* = 0.015) in the crude model. In comparison to the reference group (<4 hours) participants reporting 6 to <8 hours of sitting per day were more likely to report ever having cancer (OR 1.14, 95% CI 1.02 – 1.27, p = 0.018). Adjusting for covariates impacted considerably on the association between sitting time and cancer (*P for trend* = 0.216), and adding functional limitation to the final model further attenuated the association between sitting time and cancer (*P for trend* = 0.485).

### Heart disease

There was a crude association between sitting time and heart disease (*P for trend* <0.001). In comparison to those reporting <4 hours of sitting time per day, participants who reported 6 to <8 hours of sitting time were significantly more likely to report ever having heart disease (OR 1.13, 95% CI 1.03 – 1.23, p = 0.007). Once covariates including BMI were added to the model, the association was attenuated and the odds of ever having heart disease appeared to be slightly higher for participants in all sitting categories above the reference category (*P for trend* = 0.104), with those reporting 6 to <8 hours of sitting per day being significantly more likely to report ever having heart disease (AOR 1.13, 95% CI 1.02 – 1.25, p = 0.017). Adding functional limitation to the model attenuated the odds of ever having heart disease further (*P for trend* = 0.259), and there were no significant associations observed for any sitting time categories.

### Diabetes

The likelihood of reporting diabetes increased with increasing sitting time across all three models. Compared with those reporting <4 hours of sitting time, participants reporting 4 to <6 (OR 1.12, 95% CI 1.03 – 1.23, p = 0.010), 6 to <8 (OR 1.19, 95% CI 1.08 – 1.31, p <0.001), and ≥8 hours of sitting time per day (OR 1.15, 95% CI 1.06 – 1.25, p = 0.001) were significantly more likely to report ever having diabetes. (*P for trend* = 0.002). In the partially adjusted model, a linear increase in odds was observed between increasing sitting time and diabetes (*P for trend* <0.001), and males in the two highest quartiles of sitting time were significantly more likely to report ever having diabetes (AOR 1.18, 95% CI 1.05 – 1.32, p = 0.004; AOR 1.25, 95% CI 1.13 – 1.38, p <0.001). The association between sitting and diabetes was attenuated slightly after functional limitation was added in the final model (*P for trend* <0.001). Males reporting higher amounts of sitting (6 to <8 hours and ≥8 hours) were, however, significantly more likely to report ever having diabetes (AOR 1.15, 95% CI 1.03 – 1.28, p = 0.016; AOR 1.21, 95% CI 1.09 – 1.33, p <0.001, respectively).

### High blood pressure

The likelihood of having high blood pressure was higher, albeit slightly, for participants in each sitting category above the reference category of <4 hours (*P for trend* = 0.008) and participants reporting 4 to <6 (OR 1.06, 95% CI 1.01 – 1.11, p = 0.027) and 6 to <8 hours of sitting (OR 1.10, 95% CI 1.04 – 1.16, p = 0.001) were significantly more likely to report ever having high blood pressure than those reporting <4 hours. After partial adjustment for covariates, the likelihood of reporting high blood pressure increased linearly with increasing hours of sitting time (*P for trend* = 0.026), being highest for participants reporting ≥8 hours of sitting per day (AOR 1.09, 95% CI 1.03 – 1.15, p = 0.005). Adding functional limitation in the fully adjusted model weakened the association (*P for trend* = 0.183), but participants reporting ≥8 hours of sitting per day were significantly more likely to report ever having high blood pressure (AOR 1.06, 95% CI 1.00 – 1.12, p = 0.046).

## Discussion

Physical activity tends to decrease with age [34,35] while sitting time tends to increase [36], and the association between ageing and chronic disease has been well established at the population level [37,38]. Our findings show that, even when age was held constant, higher volumes of sitting were associated with increased odds of diabetes and overall chronic disease.

We controlled for several factors that could potentially confound the associations examined in this analysis – one of the more influential factors being functional limitation. Advancements in medical care and technology have resulted in improved treatment and earlier diagnosis of conditions, leading to a potential reduction in disease-related functional limitation [39,40]. The presence of chronic disease, however, often brings with it some decline in functional limitation [41,42]. Individuals who experience higher degrees of limitation may be less likely to participate in physical activity, and may consequently spend more time in sedentary behaviours such as sitting. Adding functional limitation in the final model allowed us to explore the unique contribution of this potentially confounding variable, while still controlling for additional covariates.

After partially adjusting for covariates including age and BMI, the associations between sitting time and most chronic disease variables included in this analysis were attenuated, with the exception of overall chronic disease and diabetes, where the associations were strengthened. After adjusting for all covariates including functional limitation, these associations were consistently attenuated, although higher quantities of sitting time were associated with significantly greater odds of having diabetes. These findings support those from a prospective cohort study by Hu et al., [12] who found that sedentary behaviour was directly associated with diabetes risk, although this earlier study focused primarily on television viewing as a marker of sedentary behaviour. Additionally, males in all sitting time categories above the reference category were significantly more likely to report ever having a chronic disease (overall chronic disease).

In response to the relatively limited body of evidence surrounding the health of Australian males, the National Male Health Policy specifically outlined a priority area for building a strong evidence base on male health [1]. The findings of this study contribute to this body of evidence by highlighting the importance of considering both physical activity and sitting time as independent factors associated with diabetes in a sample of middle-aged Australian males. Our findings suggest that sitting time is significantly associated with diabetes and overall chronic disease, independent of physical activity; building upon existing literature in which physical activity and aspects of sedentary time have been previously established as independent risk factors for CVD, metabolic syndrome, and all-cause mortality [15-18].

When interpreting the findings of this study, potential limitations must be considered. Being cross-sectional in nature, we cannot establish whether the volume of sitting time led to the development of these chronic diseases, or whether the presence of these chronic diseases influenced participants’ sitting time. Evidence from previous epidemiological studies, however, suggests that higher volumes of sitting time can present risk for diabetes [12,43]. Additional research in this particular population group is required to establish temporal sequence and further examine the potential dose–response relationships identified between sitting time and chronic diseases in the male population. Second, the self-report nature of measures used in the 45 and Up Study baseline questionnaire must be considered when interpreting the findings. Although self-report data can be affected by recall bias, or under- or over-reporting [44], self-report methods are often used in large-scale studies such as the 45 and Up Study due to the associated feasibility, cost-effectiveness, and ability to collect data from large groups of people [45].

The potential for misclassification of the variables used in this analysis must be acknowledged. It is possible that some participants may have incorrectly reported (or failed to report) having a chronic disease, while others may have under- or over-reported their daily sitting time. While these potential misclassifications may have impacted upon the strength of the observed associations, even after adjusting for a range of covariates, sitting time was still strongly and significantly associated with diabetes. In addition, while other studies on the association between sedentary time and health outcomes have adjusted for potential confounders including light intensity activity or overall energy expenditure [46,47], only data on overall time spent in physical activity in the previous week were included in this study. It is also possible that a proportion of the moderate intensity activity reported in the 45 and Up Study baseline questionnaire was actually light intensity, which may have led to an overestimation of moderate intensity physical activity. The third potential limitation is that the sitting time variables did not delineate specific domains of sitting time, such as office work, driving, other passive travel, and sitting during leisure time.

Given the potential for selection bias, as well as the 18% response rate pertaining to the 45 and Up Study and the fact that we further excluded males for whom certain data were not available, the potential impact upon the external validity of these findings should be considered. Although the characteristics of the 63,048 males included in this analysis may not be truly representative of the NSW middle-aged male population, sitting time, within this large sample of males, was strongly and significantly associated with diabetes, and to a lesser extent, overall chronic disease. Furthermore, the 45 and Up Study is the largest study of healthy ageing to be carried out in the Southern Hemisphere, and is likely to be one of the more representative large-scale cohort studies conducted globally [48].

There are also several other noteworthy strengths of this study, including the large sample size and the broad range of health-related variables on which data were collected. A total of 63,048 males from the 45 and Up Study baseline dataset were included in the analysis for the current study. Being that middle-aged males are a relatively understudied population group, the findings of his study will help to partially fill a current gap in the literature concerned with male health. This study is among the first to examine the associations between a range of chronic diseases and sitting time in middle-aged Australian males, while statistically controlling for likely confounders. The 45 and Up Study will collect much-needed longitudinal data on middle-aged and older Australian adults over the coming years, allowing researchers to monitor and investigate trends observed in this initial baseline data.

## Conclusions

It has been established that physical activity and sedentary time can be independent factors that are associated with a range of health outcomes [11,15-18]. The purpose of this study was to examine the association between sitting time and a range of chronic diseases in a sample of middle-aged Australian males, while controlling for a range of covariates.

Independent of physical activity, BMI, and additional covariates, sitting time was significantly associated with diabetes and overall chronic disease in this sample of Australian males. The findings of this cross-sectional study support and build upon previous findings while also shedding light on a relatively understudied population group in Australia. As the Australian population ages, and chronic diseases become more prevalent, it is imperative that health professionals and policy makers consider the underlying factors influencing these conditions. Self-report measures such as those used in the 45 and Up Study provide estimates of time spent in specific behaviours, such as physical activity and sedentary time. In accordance with recommendations by Healy et al. [18], however, prospective studies or well-designed intervention trials utilising objective measurement tools to assess these behaviours are needed to more clearly understand the strength, direction and temporal sequence in the association with chronic disease. Although there may be additional underlying factors influencing the development of chronic disease, as well as reported sitting time and physical activity, our findings suggest that sitting time is a distinct lifestyle factor that may be considered in efforts to decrease chronic disease in middle-aged Australian males. In addition to promoting physically active lifestyles, health promotion initiatives targeting males should also consider encouraging reductions in daily sitting time.

## Abbreviations

OR: Odds ratio; AOR: Adjusted odds ratio; CI: Confidence interval; CVD: Cardiovascular disease; CHD: Coronary heart disease; LTPA: Leisure-time physical activity; BMI: Body mass index; NSW: New South Wales; UWS: University of Western Sydney; AAS: Active australia survey; WHO: World health organization; MOS-PF: Medical outcomes study physical functioning scale; IPAQ: International physical activity questionnaire; SD: Standard deviation.

## Competing interests

The authors declare that they have no competing interests.

## Authors’ contributions

ESG conceived of the study, participated in the design and coordination of the study, performed the statistical analysis and drafted the manuscript. RRR participated in the design and coordination of the study, assisted with the statistical analysis and contributed to the preparation of the manuscript. GSK conceived of the study, participated in the design and coordination of the study and contributed to the preparation of the manuscript. All authors read and approved the final manuscript.
